# Deep learning-based segmentation of multisite disease in ovarian cancer

**DOI:** 10.1186/s41747-023-00388-z

**Published:** 2023-12-07

**Authors:** Thomas Buddenkotte, Leonardo Rundo, Ramona Woitek, Lorena Escudero Sanchez, Lucian Beer, Mireia Crispin-Ortuzar, Christian Etmann, Subhadip Mukherjee, Vlad Bura, Cathal McCague, Hilal Sahin, Roxana Pintican, Marta Zerunian, Iris Allajbeu, Naveena Singh, Anju Sahdev, Laura Havrilesky, David E. Cohn, Nicholas W. Bateman, Thomas P. Conrads, Kathleen M. Darcy, G. Larry Maxwell, John B. Freymann, Ozan Öktem, James D. Brenton, Evis Sala, Carola-Bibiane Schönlieb

**Affiliations:** 1 Department, of Applied Mathematics and Theoretical Physics, University of Cambridge, Cambridge, UK; 2 Department of Radiology, University of Cambridge, Box 218, Cambridge Biomedical Campus, Cambridge, CB2 0QQ UK; 3 Department for Diagnostic and Interventional Radiology and Nuclear Medicine, University Hospital Hamburg-Eppendorf, Hamburg, Germany; 4 jung diagnostics GmbH, Hamburg, Germany; 5 Cancer Research UK Cambridge Centre, University of Cambridge, Cambridge, UK; 6 Department of Information and Electrical Engineering and Applied Mathematics, University of Salerno, Fisciano, Italy; 7 Department of Medicine, Danube Private University, Krems, Austria; 8 Department of Biomedical Imaging and Image-Guided Therapy, Medical University Vienna, Vienna, Austria; 9 Cancer Research UK Cambridge Institute, University of Cambridge, Cambridge, UK; 10 Department of Oncology, University of Cambridge, Cambridge, UK; 11 Department of Radiology and Medical Imaging, County Clinical Emergency Hospital, Cluj-Napoca-Napoca, Romania; 12 Department of Radiology, Tepecik Training and Research Hospital, Izmir, Turkey; 13 Department of Radiology, Iuliu Hațieganu University of Medicine and Pharmacy, Cluj-Napoca-Napoca, Romania; 14 Department of Medical-Surgical and Translational Medicine-Radiology Unit, Sapienza University of Rome, Sant’Andrea Hospital, Rome, Italy; 15 Department of Clinical Pathology, Barts Health NHS Trust, London, UK; 16 Department of Radiology, Barts Health NHS Trust, London, UK; 17 Duke University Medical Center, Durham, NC USA; 18 Departmant of Obstetrics and Gynecology, Division of Gynecologic Oncology, Ohio State University Comprehensive Cancer Center, Ohio State University College of Medicine, Columbus, OH USA; 19 Department of Obstetrics and Gynecology, Gynecologic Cancer Center of Excellence, Walter Reed National Military Medical Center, Uniformed Services University of the Health Sciences, Bethesda, MD USA; 20 The John P. Murtha Cancer Center, Walter Reed National Military Medical Center, Uniformed Services University, Bethesda, MD USA; 21 Department of Obstetrics and Gynecology, Inova Fairfax Medical Campus, Falls Church, VA USA; 22 Inova Center for Personalized Health, Inova Schar Cancer Institute, Falls Church, VA USA; 23 Cancer Imaging Informatics Lab, Frederick National Laboratory for Cancer Research, Frederick, MD USA; 24 Department of Mathematics, KTH Royal Institute of Technology, Stockholm, Sweden; 25 Dipartimento Di Scienze Radiologiche Ed Ematologiche, Universita Cattolica del Sacro Cuore, Rome, Italy; 26 Dipartimento Diagnostica Per Immagini, Radioterapia Oncologica Ed Ematologia, Policlinico Universitario A. Gemelli IRCCS, Rome, Italy

**Keywords:** Deep learning, Omentum, Ovarian Neoplasms, Tomography (x-ray computed), Pelvis

## Abstract

**Purpose:**

To determine if pelvic/ovarian and omental lesions of ovarian cancer can be reliably segmented on computed tomography (CT) using fully automated deep learning-based methods.

**Methods:**

A deep learning model for the two most common disease sites of high-grade serous ovarian cancer lesions (pelvis/ovaries and omentum) was developed and compared against the well-established “no-new-Net” framework and unrevised trainee radiologist segmentations. A total of 451 CT scans collected from four different institutions were used for training (*n* = 276), evaluation (*n* = 104) and testing (*n* = 71) of the methods. The performance was evaluated using the Dice similarity coefficient (DSC) and compared using a Wilcoxon test.

**Results:**

Our model outperformed no-new-Net for the pelvic/ovarian lesions in cross-validation, on the evaluation and test set by a significant margin (*p* values being 4 × 10^–7^, 3 × 10^–4^, 4 × 10^–2^, respectively), and for the omental lesions on the evaluation set (*p* = 1 × 10^–3^). Our model did not perform significantly differently in segmenting pelvic/ovarian lesions (*p* = 0.371) compared to a trainee radiologist. On an independent test set, the model achieved a DSC performance of 71 ± 20 (mean ± standard deviation) for pelvic/ovarian and 61 ± 24 for omental lesions.

**Conclusion:**

Automated ovarian cancer segmentation on CT scans using deep neural networks is feasible and achieves performance close to a trainee-level radiologist for pelvic/ovarian lesions.

**Relevance statement:**

Automated segmentation of ovarian cancer may be used by clinicians for CT-based volumetric assessments and researchers for building complex analysis pipelines.

**Key points:**

• The first automated approach for pelvic/ovarian and omental ovarian cancer lesion segmentation on CT images has been presented.

• Automated segmentation of ovarian cancer lesions can be comparable with manual segmentation of trainee radiologists.

• Careful hyperparameter tuning can provide models significantly outperforming strong state-of-the-art baselines.

**Graphical Abstract:**

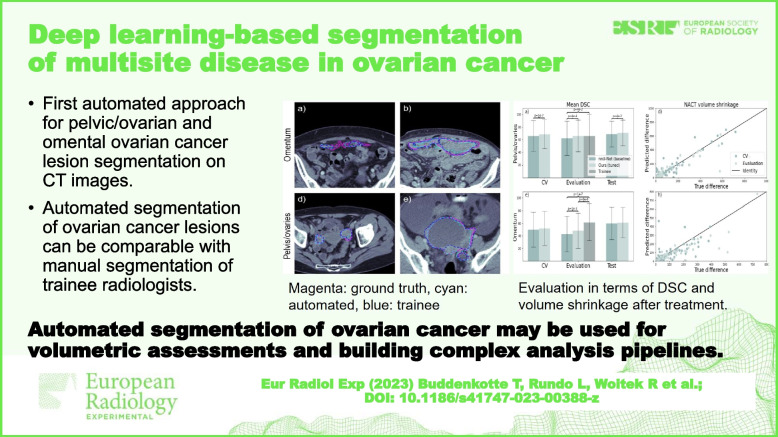

**Supplementary Information:**

The online version contains supplementary material available at 10.1186/s41747-023-00388-z.

## Background

After decades of unchanged treatment regimens for patients with ovarian cancer and little improvement in patients’ survival, this treatment landscape is currently changing, and an increasing number of therapeutic options can be offered. For standard treatments with chemotherapy, the interpretation of oncological computed tomography (CT) scans by an expert radiologist for evaluation of tumour spread usually includes only a small number of one- or two-dimensional lesion measurements that typically follow response evaluation criteria in solid tumours guidelines (RECIST 1.1) [1]. However, these measurements are subjective, lack sensitivity for the early detection of treatment response and progression [2], and show only limited correlation with patient outcomes [3, 4]. Novel treatments like immunotherapy, for example, require dedicated guidelines [5] and often several months of treatment monitoring before pseudo-progression can reliably be distinguished from response. The development of non-invasive imaging biomarkers for response assessment as well as patient selection for such treatments is still in its infancy [6]. Detailed volumetric response assessment as well as radiomics have the potential to improve clinical decision making for both, patients undergoing standard-of-care chemotherapy and novel treatments. However, both require manual segmentation of the entire tumour burden by a radiologist. In advanced-stage ovarian cancer with multi-site peritoneal disease, the detailed segmentation and annotation of a single scan can become a very time-consuming task and is only done for research purposes thus omitting potentially relevant information from clinical decision-making.

Recently, deep neural networks based on the U-Net architecture [7] have shown promising results in challenging medical image segmentation problems. For example, the no-new-Net (nnU-Net) framework [8, 9] achieved state-of-the-art performance in various biomedical segmentation challenges, such as the Medical Segmentation Decathlon [10]. A recent survey also showed that nine out of ten top two performing teams in the 2020 MICCAI segmentation challenges are built using the nnU-Net framework as a baseline [11].

Deep neural networks are a promising solution for time-efficient and observer-independent segmentation of ovarian cancer lesions. Such methods allow volumetric response assessment of the disease instead of the currently used RECIST 1.1. guidelines [1] and have the potential to reduce the manual annotation time [12, 13] allowing researchers to create large-scale datasets with high-quality segmentations. Such datasets enable the creation of future disease quantification and response prediction tools to support clinical decision making and facilitate the development of clinical trials.

To the best of our knowledge, this is the first paper to propose a deep learning-based approach for the automated segmentation of the disease located in the pelvis/ovaries and omentum, which are the predominant disease sites. A recently proposed automated approach based on classical machine learning focused on perihepatic and perisplenic ovarian cancer metastases segmentation [14, 15] but did not address the most common locations.

## Methods

### Datasets

Patients were recruited prospectively into the respective studies. All images used in this study were acquired per clinical request and subsequently collected after informed patient consent was obtained for use in research approved by the local ethical review board. We retrospectively collected scans using only contrast-enhanced axial CT images from patients with high-grade serous ovarian carcinoma (HGSOC). Additional information on the acquisition settings of the computed tomography examination is given in the Supplementary Materials. The diagnosis of HGSOC was confirmed in all patients through biopsy and histopathological analysis. Patients without contrast-enhanced CT scans or with unclear histopathological diagnosis were excluded.

For this study, we had a total of 451 scans from four institutions and two countries available. As the majority of data (*n* = 380) was obtained in the UK, we decided to use this part of the data for training and evaluation of the method. In particular, the larger subset (*n* = 276) obtained at Addenbrooke’s Hospital (Cambridge University Hospitals NHS Foundation Trust, Cambridge, UK) was used for training and the remaining scans (*n* = 104) from St. Bartholomew’s Hospital (Barts Health NHS Trust, London, UK) were used for evaluation. The remaining scans (*n* = 71) obtained in the Gynecologic Cancer of Excellence programme and the Cancer Imaging Archive (https://www.cancerimagingarchive.net/) in the USA were used as a test set.

The patients across all datasets were treated with either immediate primary surgery (IPS) or three to six cycles of neoadjuvant chemotherapy (NACT). Among the 157 patients in the training dataset, 119 were treated with NACT for which both pre- and post-treatment scans were available. The remaining 38 patients were treated with IPS for which only the pre-treatment scan was available. The scans in the evaluation set were obtained from 53 patients who were treated with NACT. For all patients both pre- and post-NACT scans were available. However, two post-NACT scans were removed from the dataset as no disease was visible anymore after the treatment. All patients in the test data received IPS. Only pre-treatment scans are contained in this dataset.

**Fig. 1:**
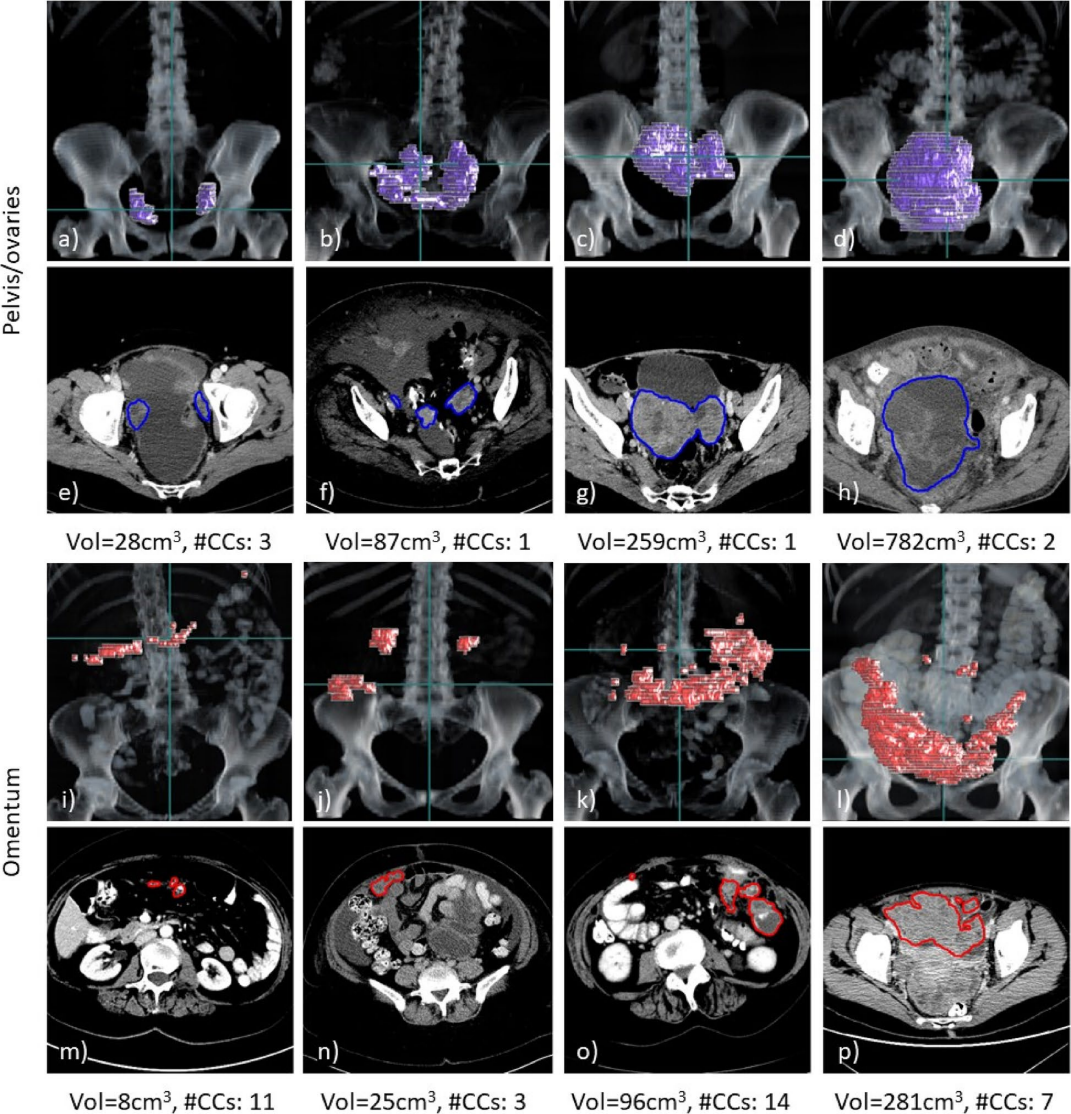
Examples of three-dimensional volume renderings (**a**–**d**, **i**–**l**) and axial slices (**e**–**h**, **m**–**p**) for pelvic/ovarian and omental lesions of high-grade serous ovarian carcinoma patients. For each example, the ground truth tumour volume (Vol) and number of connected components (#CCs) are shown. The scans shown were all contained in the training set and selected such that their lesion volume equals the 25, 50, 75, and 90 percentiles of the lesion volume in the training set (left to right). The horizontal green line in the rendering visualisations corresponds to the axial slice shown below. Both disease sites demonstrate a great variability of disease expression among different patients, which poses a great challenge for manual and automated segmentation methods

The dataset compositions, including patient age, resolution of the reconstructed images and quantitative measures of the two disease sites, are shown in Table 1, and further details describing the heterogeneity of the acquisition protocols are provided in the Supplementary Materials. Further information on the patients such as ethnicity and clinical condition were not available to us due to the anonymisation of the scans. Examples of the two disease sites are shown in Fig. 1.

**Table 1:** Composition of the three datasets (total number of scans = 451) including information on voxel spacing and disease expression along all available time points

Dataset	Training	Validation	Test
Number of scans	276	104	71
Pretreatment scans	157	53	71
Post-NACT scans	119	51	0
Patient age [years]			
Median	65.5	65.5	63
Min–max	29–90	35–85	41–80
Pixel spacing [mm]			
Median	0.68	0.76	0.77
Min–max	0.53–0.93	0.61–0.96	0.57–0.98
Slice thickness [mm]			
Median	5.0	5.0	5.0
Min–max	1.25–5.0	1.5–5.0	2.0–7.5
Pelvic/ovarian tumour			
Number of scans showing tumour in this location	246	102	69
Mean volume [cm^3^]	275	241	381
Mean number of connected components	2.4	2.6	2.4
Omental tumour			
Number of scans showing tumour in this location	198	98	56
Mean volume [cm^3^]	119	197	146
Mean number of connected components	6.7	5.3	5.7

Due to the anonymisation of the datasets, no information on the ethnicity of the patients was prevalent and the patient age was not available in 14 out of 71 patients in the test data. Additional information on the acquisition settings of the computed tomography examination is given in the Supplementary Materials. *NACT* Neoadjuvant chemotherapy

### Manual annotation

All manual segmentations were performed using the Microsoft Radiomics application (project InnerEye; https://www.microsoft.com/en-us/research/ project/medical-image-analysis/), Microsoft, Redmond, WA, USA).

All segmentations used as ground truth in this study were either manually segmented or corrected by radiologist 1 (R.W.; consultant radiologist with 10 years of experience in oncological and gynaecological imaging). The training data set was segmented solely by R.W. The evaluation data set was pre-segmented by a trainee radiologist (3 years of experience in oncological and gynaecological imaging) and subsequently reviewed and corrected by R.W. For all scans in this dataset, both the unrevised trainee and the ground truth segmentations were available. The test set was segmented by H.S. (6 years of experience in oncological and gynaecological imaging) and reviewed by R.W. All segmentations in this dataset were found to be of satisfying quality; therefore, only one set of segmentations (ground truth) was available for these scans.

### Deep learning model

As no literature exists on the segmentation of pelvic/ovarian and omental lesions in ovarian cancer, we first used the three-dimensional (3D) full-resolution U-Net of the nnU-Net framework [8, 9] as a baseline for this segmentation task. The framework automatically adapts to new datasets, suggests model hyper-parameters and can be considered the current state of the art in 3D biomedical image segmentation [10, 11]. Our model was obtained by reimplementing the nnU-Net framework from scratch, benchmarking both implementations and performing extensive hyper-parameter optimisation. As already observed by the authors of nnU-Net [8, 9], we did not find an impact of minor architectural changes on the performance of our model. Instead, we decided to focus on fine-tuning the hyper-parameters suggested by the nnU-Net framework.

As with all current state-of-the-art 3D biomedical segmentation networks, nnU-Net is based upon the U-Net architecture [7, 11]. For our dataset, nnU-Net suggested a simple U-Net with six stages, LReLU activation functions, instance normalisation and 32 filters in the first block doubling at each stage. We found it beneficial to reduce the number of stages to four and replace the encoder with a ResNet [16] of 1, 2, 6 and 3 blocks per stage. The nnU-Net framework further suggests training the networks for 250,000 batches of size 2 using an SGD optimiser with Nesterov’s momentum of factor 0.99, weight decay of 3 × 10^–5^ and a polynomial decaying learning rate from 0.01 to 0. We instead found it beneficial to increase the batch size to 4, the weight decay to 10^–4^, decrease the momentum to 0.98 and change the learning rate schedule to a linear ascent plus cosine decay with a maximum learning rate of 0.02.

The framework uses resizing of voxel spacing and *Z*-normalisation of the grey values as preprocessing and various spatial (rotation, scaling, flipping) and grey value-based (Gaussian noise, multiplicative scaling, contrast, blurring, gamma) transformations on the fly for data augmentation. During inference, a Sliding Window algorithm with Gaussian weighting and test-time augmentations by evaluating all eight permutations of flipping over *x*-, *y*- and *z*-axis was applied. We found no benefit in changing the pre-processing, data augmentation and evaluation based on the Sliding Window algorithm; hence, they were left unchanged and applied as suggested by the authors of nnU-Net [8, 9].

### Code and model availability

All code was developed using Python (version 3.9.4) as a programming language and the deep learning framework PyTorch (version 1.9.0). To make the training reproducible and share our model with the research community, we made the training code, inference code and model hyper-parameters and weights publicly available on our code GitHub repository at: https://github.com/ThomasBudd/ovseg.

### Statistical analysis

To test whether differences in DSC were significant, we computed *p* values using the Wilcoxon test on paired results. These computations were performed using the Python package SciPy.

## Results

### Performance assessment

**Fig. 2:**
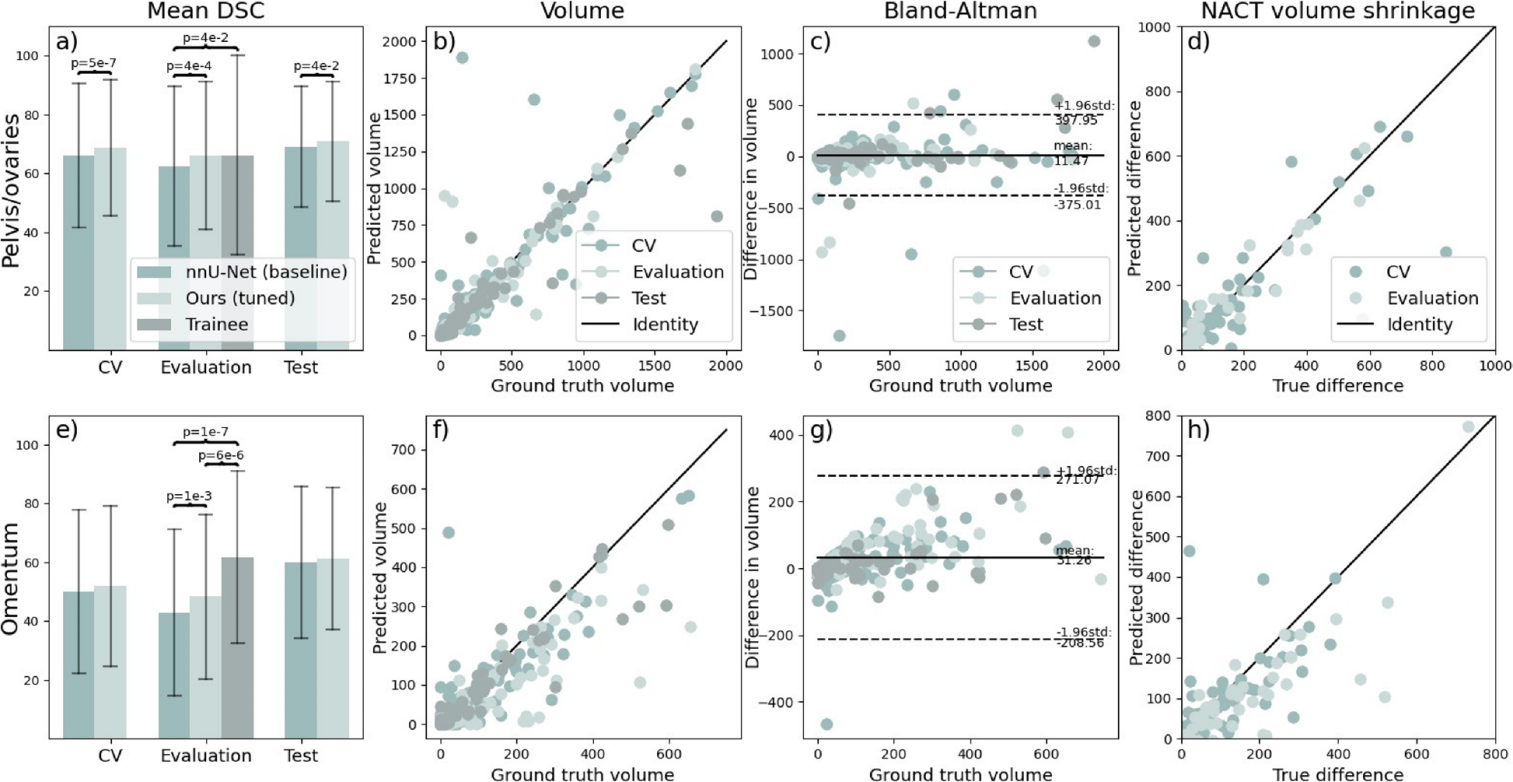
Evaluation of model performance on unseen datasets in terms of DSC (**a**, **e**) and volume (**b**–**d**, **f**–**h**). Trainee radiologist segmentations were only available on the evaluation set. The brackets indicate significant differences. All volumes are given in cm^3^. It can be observed in panels **a** and **e** that our method outperforms the nnU-Net baseline for both sites on the evaluation and test set and that our method does not perform significantly different from a trainee radiologist in segmenting pelvic/ovarian lesions in contrast to nnU-Net. Panels **b**–**d** and **f**–**h** suggest that the model in its current state can be used to determine disease volume for both sites. *DSC* Dice similarity coefficient, *nnU-Net* No-new-Net

All metrics were only computed on scans containing the corresponding disease site. The results (expressed as mean ± standard deviation) are summarised in Fig. 2 and Supplementary Table S1. Both nnU-Net and our model were trained using a five-fold cross-validation scheme ensuring that two scans of the same patient are contained in the same fold. In cross-validation, the models achieved a mean DSC of 66 *versus* 69 (*p* = 5 × 10^–7^) for the pelvic/ovarian and 50 *versus* 52 (*p* = 0.203) for the omental lesions. The mean DSC performance on the evaluation set was 62 *versus* 66 (*p* = 1 × 10^–4^) and 43 *versus* 48 (*p* = 1 × 10^–3^) for the pelvic/ovarian and omental lesions. The test set performance in terms of mean DSC was 69 *versus* 71 (*p* = 0.042) and 60 *versus* 61 (*p* = 0.123) for pelvic/ovarian and omental lesions. The statistical significance of the difference in performance was confirmed by swapping the training set with the merged evaluation and test set and repeating training and inference with results being summarised in Supplementary Table 2. We suspected that the performance discrepancy between the evaluation and test set was caused by differences in disease distribution as the patients in the evaluation set qualified for NACT and the patients in the test set for IPS as a treatment. To investigate whether this difference might have an impact on the performance, we split the training set by applied treatment and evaluated the cross-validation performance of our model on the subsets. For pelvic/ovarian lesions, the model achieved a mean DSC value of 67 (*n* = 206) *versus* 77 (*n* = 40), whereas for omental lesions of 52 (*n* = 176) *versus* 53 (*n* = 22) for the subset of patients that received NACT and IPS, respectively.

To investigate the effect of increasing training dataset size, we additionally trained three distinct networks on the full training data (*n* = 276) instead of relying on five-fold cross-validation which uses only 80% of the training data (*n* = 220) per fold and compared the results to nnU-Net. The achieved performance in terms of mean DSC was 66 (*p* = 1 × 10^–4^) and 51 (*p* = 10^–6^) on the evaluation set and 72 (*p* = 4 × 10^–3^) and 64 (*p* = 5 × 10^–3^) on the test set for pelvic/ovarian and omental lesions, respectively.

Furthermore, we compared the DSC values achieved by nnU-Net and our model (trained in five-fold cross-validation) with the trainee radiologist on the evaluation set. Both models performed significantly worse than the trainee radiologist for the segmentation of omental lesions (*p* = 1 × 10^–7^ and 6 × 10^–6^). However, considering the pelvic/ovarian lesions, nnU-Net performs significantly inferior to the trainee radiologist (*p* = 0.041), while there was no such significant difference between our proposed model and the trainee radiologist (*p* = 0.371).

Additionally, we compared the disease volume of the ground truth with the automated annotations of our proposed model. Despite the presence of some outliers, Fig. 2 shows close agreement in volume for both disease sites considering the volume comparison of individual scans (second column) and the difference of pre- and post-NACT volume. The Bland–Altman plot shows that on average the ground truth volume is greater than the predicted volume for both sites.

**Fig. 3:**
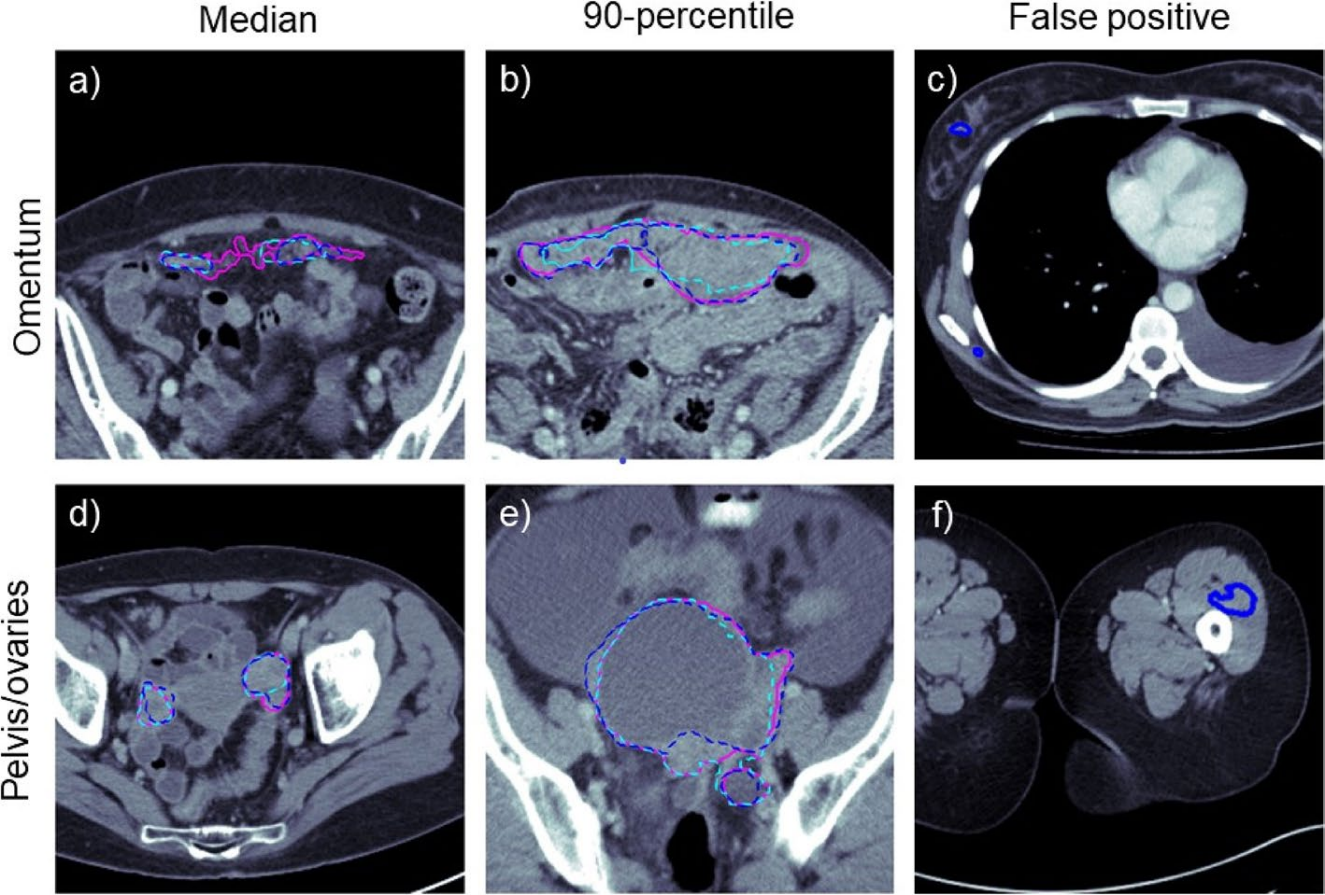
Examples of ground truth, automated and trainee radiologist segmentations (pink, cyan and blue, respectively). The first two columns (**a**, **b**, **d**, **e**) show the cases with median and 90-percentile DSC from the pooled validation and test set. The visual comparison between the automatically generated and manual trainee radiologist segmentation demonstrates typical mistakes of the two instances. Both seem to struggle with the inclusion and exclusion of objects close to the segmentation boundary. The last column (**c**, **f**) shows examples of outliers at the extreme ends of the volumes. The segmentation model confused dense components of breast tissue with omental disease as both are embedded in fat

Examples of automated and trainee radiologist segmentations are presented in the left and middle columns of Fig. 3.

### Outlier and error analysis

To assess common errors and outliers, the evaluation and test set were pooled (*n* = 175) and inspected visually and quantitatively.

Low DSC values were regularly found on scans with low ground truth volume. In the subset of scans with the bottom 25% DSC performance, lower median disease volume was found compared to the full set (omentum, 6 *versus* 21 cm^3^; pelvis/ovaries, 9 *versus* 37 cm^3^). Vice versa, on the scans with the 25% lowest volumes, lower mean DSC performance was found when compared to the full set (omentum, 35 *versus* 55; pelvis/ovaries, 46 *versus* 68).

A visual evaluation revealed common false positive predictions in the extreme ends of the scan volumes outside of anatomic regions where disease commonly occurs. On 41 scans, the omental disease was falsely annotated in breast tissue, in 13 cases near the scapulae and in five cases, false-positive annotations were found in the thighs. Examples of such false-positive annotations can be found in the right column of Fig. 3.

To better understand the influence of false-positive and false-negative predictions, we computed the sensitivity and precision. For both disease sites, the sensitivity was found to be lower than the precision (omentum, 52 *versus* 70; pelvis/ovaries, 69 *versus* 73).

Another source of error was the confusion between classes. We found that in 12 out of 170 cases containing pelvic/ovarian disease, at least parts of the ground truth annotation intersected with automated annotation of the omental disease. Vice versa in 18 out of 155 scans containing omental disease at least one voxel of omental disease was marked as pelvic/ovarian disease by the algorithm. Even to the trained radiologist’s eye, it can be challenging to distinguish between extensive pelvic and omental disease when tumours form conglomerates.

## Discussion

This work presents the first automated deep learning-based ovarian cancer CT segmentation approach for the two main disease sites: the pelvis/ovaries and the omentum, while previous work only addressed less common disease sites [14, 15]. While the relatively low DSC values suggest inferior performance compared to expert consultant radiologists, we could demonstrate similar performance to a trainee radiologist with 3 years of experience for pelvic/ovarian lesions despite the complexity of the disease and using only a few hundred scans for training. Moreover, we demonstrated that our model significantly outperforms the well-established state-of-the-art framework nnU-Net for the segmentation of the pelvic/ovarian lesions and generalises to a test set from a different country. The heterogeneity of the disease between patients was demonstrated in Fig. 1. This causes great challenges for both manual and automated segmentation and might be the main reason for the performance gap between our model and the ground truth. Furthermore, a gap in performance for the segmentation of omental disease was found between the trainee radiologist and the proposed method. This might be due to the difficulty of the segmentation task as omental disease often exhibits poor contrast and irregular shape and sparse deposits. Our analysis also suggested that the proposed model might perform better on scans of patients treated with IPS (*n* = 38 in cross-validation and test set) compared to those treated with NACT (*n* = 238 in cross-validation and evaluation set). Another reason for the performance differences between the evaluation and test set might be differences in patient stratification between the two different countries of origin (UK *versus* USA).

We believe that the model in its current form is already of clinical relevance. Previous approaches have already demonstrated that deployed deep learning models can decrease the manual preparation time in clinical routines [12] and in research settings for the creation of large-scale datasets [13]. These datasets might ultimately allow the creation of sophisticated chemotherapy response or survival prediction models [17] and improve patient care. Further, Fig. 2 suggests that the model might be ready to allow volumetric assessment of the disease without requiring manual interventions. This might be of particular interest to centres without specialisation in high-grade serous ovarian cancer.

Our main limitations are the following. The difference between sensitivity and precision, which was especially large for the omental lesions, indicates that the DSC can be further improved by careful calibration of the model parameters as suggested in [18], which were not exhaustively included in this work due to limitations of the computational budget. This might also be a solution for underestimation of disease volume as shown in the Bland–Altman plots of Fig. 2. The false positive predictions in the extreme ends of the scans, such as the breast tissue and the lower limbs, might be removed in two different ways. Firstly, organ segmentation [13] can be applied to identify the regions in which the lesions typically occur. Secondly, the patch sampling scheme can be modified as the currently employed schemes undersample the extreme ends of the volumes. Next, combining the proposed convolutional neural network model with other models such as ones based on transformers or tumour-sensitive matching flow can be attempted to improve the performance. For example, a recently introduced vision transformer-based framework has shown lower performance when being compared against nnU-Net but could demonstrate that the ensembling of both methods outperformed the standalone frameworks [19]. In addition, future approaches should integrate the other disease sites of HGSOC into automated segmentation approaches. However, those that occur less frequently are on average of lower volume and often spread throughout the whole abdomen and beyond, thus imposing challenges for automated segmentation models. For the training of future models, it is also desirable to have access to larger datasets with high-quality annotations. This was not available to us in this initial study as annotations are time-consuming to obtain and experts for this disease are rare. We believe that larger datasets along with continuously exploring new training methods will help close the performance gap between the consultant radiologists and the deep learning model. Furthermore, we plan to extensively test more variations in architecture, such as network scale or using transformers or novel convolutional residual blocks, and hyper-parameters, such as augmentation methods and loss functions, with the goal of obtaining an even better performing automated segmentation model.

To summarise, we presented the first deep learning-based approach for ovarian cancer segmentation on CT and the first automated approach for the segmentation of pelvic/ovarian and omental lesions and demonstrated both state-of-the-art performance, as well as common errors of our method.

### Supplementary Information

**Additional file 1:** **Supplementary Table 1.** Model and trainee performance on unseen datasets in terms of DSC (mean ± std). Results on the training set were computed using the cross-validation predictions, thus no scores are available for the model trained on 100% of the training data. Significant differences compared to nnU-Net and the trainee our model and the baseline and the trainee and our model are marked with the symbols * and an §, respectively. Trainee radiologist segmentations were only available on the evaluation set. Our implementation is publicly available at https://github.com/ThomasBudd/ovseg. **Supplementary Table 2.** Model and trainee performance on unseen datasets in terms of DSC (mean ± std) obtained by swapping the training set with the evaluation and test set. Significant differences compared to nnU-Net and the trainee are marked with the symbols * and an §, respectively. Trainee radiologist segmentations were only available on the evaluation set. Our implementation is publicly available at https://github.com/ThomasBudd/ovseg. **Supplementary Figure 1.** Comparison of the model’s performance in terms of DSC on scanners from different manufacturers. **Supplementary Figure 2.** Training and validation curve over the course of one full training. Each epoch was defined as 250 training batches. The validation error was estimated by aggregating the loss of 25 batches.

## Data Availability

Twenty scans of the test data were collected from the cancer imaging arxiv (https://www.cancerimagingarchive.net/). All remaining data used in this study is hospital proprietary data and not publicly available. The code and models are publicly available at https://github.com/ThomasBudd/ovseg.

## Abbreviations

3D: Three-dimensional
CT: Computed tomography
DSC: Dice similarity coefficient
HGSOC: High-grade serous ovarian carcinoma
IPS: Immediate primary surgery
NACT: Neoadjuvant chemotherapy
nnU-Net: No-new-Net
