# Supplementary material for: Deep learning-based segmentation of multisite disease in ovarian cancer
Source: Eur Radiol Exp. 2023 Dec 7;7:77. doi: 10.1186/s41747-023-00388-z (PMC10700248; doi:10.1186/s41747-023-00388-z)
Supplement: Supplementary file 1 — Additional file 1: Supplementary Table 1. Model and trainee performance on unseen datasets in terms of DSC (mean ± std). Results on the training set were computed using the cross-validation predictions, thus no scores are available for the model trained on 100% of the training data. Significant differences compared to nnU-Net and the trainee our model and the baseline and the trainee and our model are marked with the symbols * and an §, respectively. Trainee radiologist segmentations were only available on the evaluation set. Our implementation is publicly available at https://github.com/ThomasBudd/ovseg. Supplementary Table 2. Model and trainee performance on unseen datasets in terms of DSC (mean ± std) obtained by swapping the training set with the evaluation and test set. Significant differences compared to nnU-Net and the trainee are marked with the symbols * and an §, respectively. Trainee radiologist segmentations were only available on the evaluation set. Our implementation is publicly available at https://github.com/ThomasBudd/ovseg. Supplementary Figure 1. Comparison of the model’s performance in terms of DSC on scanners from different manufacturers. Supplementary Figure 2. Training and validation curve over the course of one full training. Each epoch was defined as 250 training batches. The validation error was estimated by aggregating the loss of 25 batches. [file 41747_2023_388_MOESM1_ESM.docx]

**Supplementary Materials**

**Acquisition Characteristics of the Analyzed Ovarian Cancer CT Datasets**

There is high variation in image acquisition parameters and scanner manufacturers across all three datasets. In the training data, the most used scanner manufacturer was Siemens followed by GE, Philips, and Toshiba with 204, 56, 12 and 4 scans, respectively. In this dataset, one, 45, 115, 110 and 5 scans were acquired at a KVP of 80, 100, 120, 130 and 140 respectively. In the validation data, the most used scanner manufacturer was Siemens followed by Toshiba, Philips, and GE with 64, 24, 8 and 8 scans, respectively. Here all scans were acquired at a KVP of 120, except one for which 100 KVP was used. In the test dataset, 38 scans were acquired using GE scanners, followed by 26 Siemens, four Toshiba, one Philips, one Imatron, and one MPTronic scanner. In this dataset eight, 58, and five scans were acquired at 100, 120, 130 KVP respectively.

We analyzed the influence of the acquisition protocol on the performance of the model in terms of DSC. Therefore, we pooled the validation and test set. As the resulting dataset only showed large heterogeneity in terms of manufacturer, but not in terms of KVP we decided to focus only on the influence of the manufacturer to the performance and removed the two scans acquired my Imatron and MPTronic scanners from the dataset due to low sample size. Supplementary Figure 1 shows the manufacturer of the CT scanner versus the performance of the model in terms of DSC. It can be observed that differences are difficult to spot by eye due to the high variability of the DSC. We further compared each manufacturer against the remaining ones using the Wilcoxon Rank test. For the pelvic/ovarian disease we computed the p-values 0.296 (GE Medical Systems), 0.527 (Siemens), 0.743 (Toshiba), and 0.244 (Philips). For omental disease the p-values were computed as 0.625 (GE Medical Systems), 0.292 (Siemens), 0.043 (Toshiba), and 0.887 (Philips).

To summarize, in all except one case no significantly different performance in dependence of the scanner manufacturer could be found.

**
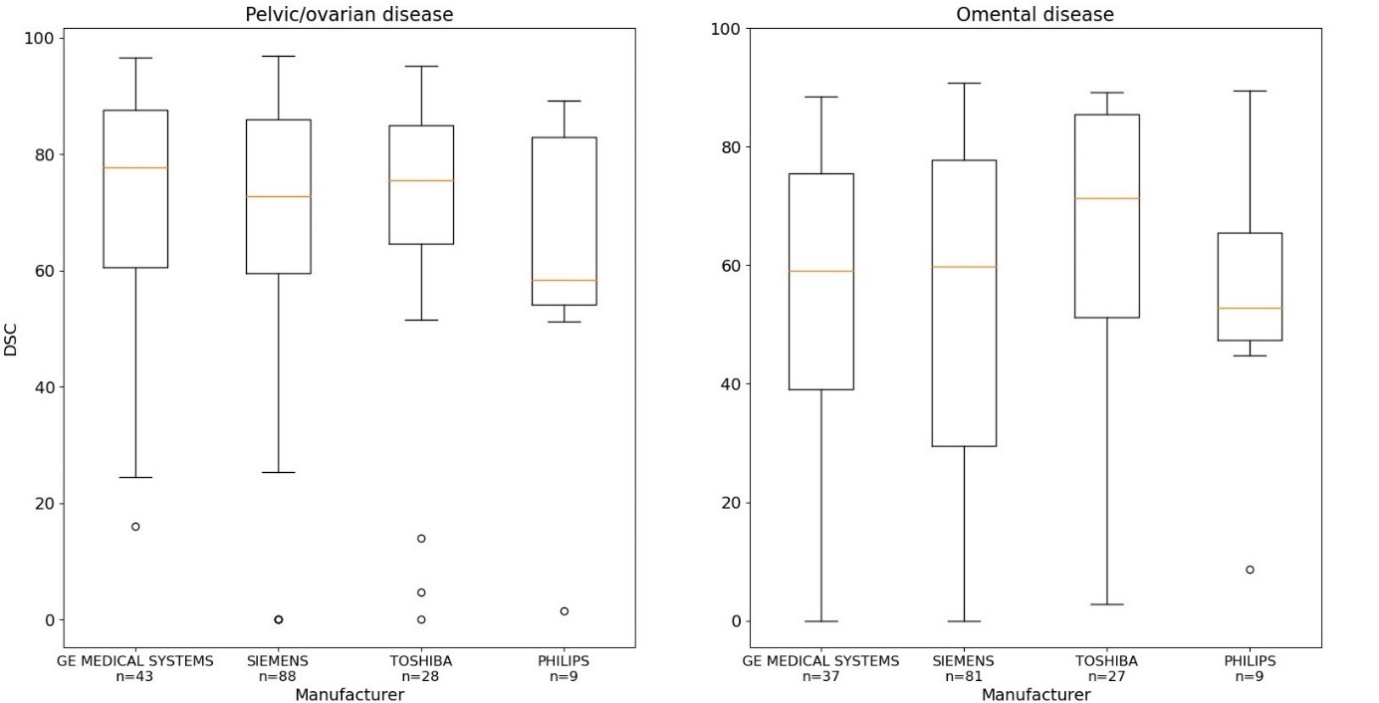
**

**Supplementary Figure 1.** Comparison of the models performance in terms of DSC on scanners from different manufacturers.

**Hyper-parameter tuning**

In the first step of our hyper-parameter tuning we reduced the in-plane resolution of the scans from 0.67 mm (as suggested by nnU-Net) to 0.8mm, which later allowed us to reduce the patch size. We also applied progressive learning by splitting the training into four quarters and reducing both the number of voxels per sample and the magnitude of the grey value augmentations by a factor of four, three and two over the first three quarters of the training. The last quarter was applied as usual.

Next, we applied several architectural modifications, but only found increase the capacity of the network to by replacing the decoder with a ResNet to be beneficial. In detail we changed the six stage U-Net (i.e. a U-Net with five downsampling operations in the decoder) to a four stage U-Net with 1, 2, 6 and 3 residual blocks in the corresponding stages. We used standard residual blocks where each block consisted of two convolution-Instance normalization-LReLU units followed by a skip connection. As now the number of downsampling operations was reduced from five to three we could reduce the patch size from 224 to 216 which reduced the training time again.

Lastly, we found a great improvement by altering the convergence parameters. We found a benefit in changing the learning rate schedule from the almost linear decay from 0.01 to 0 as suggested by nnU-Net to a linear ascent plus cosine decay with maximum learning rate 0.02. The increase in maximum learning rate was not possible without using the linear warmup, which happened over the first five percent of the training. Next, we made use of the full 24GB of VRAM in our GPUs and increased the batch size from two to four. Initially this caused a performance decrease, but we finally obtained an increase in performance when decreasing the momentum factor of the SGD from 0.99 to 0.98 and increasing the weight decay from 3x10-5 to 10-4. A typical training curve of one of the models trained in four-fold CV can be seen in Supplementary Figure 2. The two jumps in loss after epoch 250 and 500 are due to the progressive learning as here the input patch size and resolution were increased. During inference the mean of the softmax-outputs of allmodels was used as a final prediction.


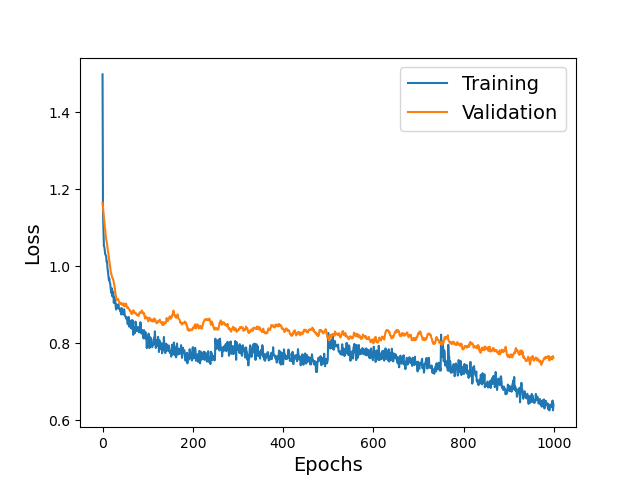


**Supplementary Figure 2.** Training and validation curve over the course of one full training. Each epoch was defined as 250 training batches. The validation error was estimated by aggregating the loss of 25 batches.

|  | Training | | Evaluation | | Test | |
| --- | --- | --- | --- | --- | --- | --- |
| Method | Pelvis/  ovaries | Omentum | Pelvis/  ovaries | Omentum | Pelvis/  ovaries | Omentum |
| nnU-Net | 66±25 | 50±28 | 62±27^§^ | 43±28^§^ | 69±21 | 60±26 |
| Ours CV | 69±23^*^ | 52±27 | 66±25^*^ | 48±28^§^ | 71±20^*^ | 61±24 |
| Ours 100% |  |  | 66±26^*^ | 51±27^§^ | 72±19^*^ | 64±25^*^ |
| Trainee |  |  | 66±34^*^ | 62±29^*^ |  |  |

**Supplementary Table 1.** Model and trainee performance on unseen datasets in terms of DSC (mean ± std). Results on the training set were computed using the cross-validation predictions, thus no scores are available for the model trained on 100% of the training data. Significant differences compared to nnU-Net and the trainee are marked with the symbols * and §, respectively. Trainee radiologist segmentations were only available on the evaluation set. Our implementation is publicly available at https://github.com/ThomasBudd/ovseg.

**Results with swapped training and evaluation/test data**

To confirm our findings we repeated the training inference of the five-fold cross-validation models by swapping the training set with the merged evaluation and test set. The results in terms of mean ± std DSC are shown in Supplementary Table 2. As before, our approach outperformed nnU-Net significantly for the pelvic/ovarian segmentation on all datasets and for the omental lesions on the (original) evaluation set. In comparison to the previous experiments it can be observed that the performance on the (original) evaluation and test sets increased and decreased on the (original) training set. This is not surprising as the results on the (original) evaluation and test set were obtained in cross-validation which implies that patients from the same cohort have been part of the training set during these experiments. Due to this increase in terms of mean DSC, it can be observed that the nnU-Net and our model no longer perform significantly worse than the trainee radiologist on the evaluation set.

| Swapped | (original) Training | | (original) Evaluation | | (original) Test | |
| --- | --- | --- | --- | --- | --- | --- |
| Method | Pelvis/  ovaries | Omentum | Pelvis/  ovaries | Omentum | Pelvis/  ovaries | Omentum |
| nnU-Net | 63±26 | 44±31 | 66±25 | 54±28^§^ | 72±19 | 59±29 |
| Ours CV | 65±25* | 45±29 | 70±21* | 60±25* | 74±17* | 61±27 |
| Trainee |  |  | 66±34 | 62±29* |  |  |

**Supplementary Table 2**. Model and trainee performance on unseen datasets in terms of DSC (mean ± std) obtained by swapping the training set with the evaluation and test set. Significant differences compared to nnU-Net and the trainee are marked with the symbols* and ^§^, respectively. Trainee radiologist segmentations were only available on the evaluation set. Our implementation is publicly available https://github.com/ThomasBudd/ovseg.
